# Protein Engineering with Biosynthesized Libraries from *Bordetella bronchiseptica* Bacteriophage

**DOI:** 10.1371/journal.pone.0055617

**Published:** 2013-02-07

**Authors:** Tom Z. Yuan, Cathie M. Overstreet, Issa S. Moody, Gregory A. Weiss

**Affiliations:** 1 Department of Molecular Biology and Biochemistry, University of California Irvine, Irvine, California, United States of America; 2 Department Chemistry, University of California Irvine, Irvine, California, United States of America; Centro Nacional de Biotecnologia - CSIC, Spain

## Abstract

Phage display offers a powerful approach to engineer protein affinity. A naturally occurring analog to phage display, the *Bordetella bronchiseptica* bacteriophage (BP) employs a highly variable protein termed the major tropism determinant (Mtd) to recognize its dynamic host. Propagation of BP provides a self-made phage library (SMPL) with vast numbers of phage particles, each displaying a single Mtd variant. We report applying the diversity of the BP-SMPL to access a tyrosine-rich library of Mtd variants. Expression of the SMPL-engineered Mtd variant as a GST-bound fusion protein demonstrated specific binding to the target T4 lysozyme with dissociation constants in the sub-micromolar range. The results guide future experiments with SMPLs applied to protein engineering.

## Introduction

*In vitro* molecular evolution is extensively used for the identification and optimization of binding by receptors and biopharmaceuticals [1], [2], [3], [4]. Such experiments take cues from the immune system, and offer rapid evolution of high affinity binding proteins. *Bordetella bronchiseptica* bacteriophage (BP) have evolved a diversity generating retroelement, which synthesizes self-made phage libraries (SMPLs) by introducing DNA mutations into the gene encoding the major tropism determinant (Mtd) protein on the tail fibers of each BP [5], [6]. The BP-SMPLs demonstrate various attributes found in effective molecular display systems - vast diversity, flexible binding to a range of targets, and encapsulated sequence information.

**Figure 1:**
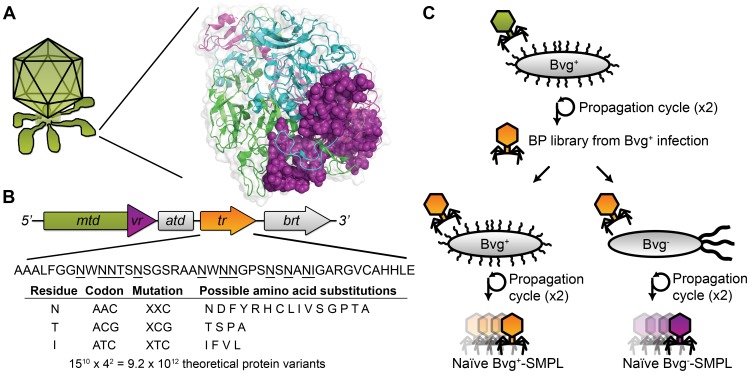
Mtd protein structure and SMPL diversity generation. (A) *Bordetella* phage expresses six distal tail fibers, with the Mtd protein located at the end of each tail fiber. The Mtd structure consists of a homotrimer found on the tail fibers of the BP (PDB: 1YU4) [25]. During SMPL formation, the variable region (VR) of the trimer (purple spheres) is diversified, and is responsible for determining binding specificity to target proteins on the surface of *Bordetella bronchiseptica* [25], [26]. (B) This schematic gene map of the diversity generating retroelement identifies mutable codons within the *tr*. The targeted codons include adenines outside the wobble position, and encode the underlined amino acids to generate 9.2×10^12^ variants of the Mtd protein. (C) Generation of the BP-SMPLs through phage production by infecting Bvg^+^ *Bordetella bronchiseptica*, followed by propagation in either Bvg^+^ or Bvg**^−^** phase *Bordetella* strains.

The Mtd protein also determines viral specificity for its host, *B. bronchiseptica*, by binding to outer membrane proteins expressed on the bacterial surface [7]. The bacterial host alters its surface proteins during transitions between virulent (Bvg^+^) and avirulent (Bvg**^−^**) phases of its life cycle. *Bordetella* bacteria exist primarily in this bimodal phase system. Activation of the *Bordetella* virulence control locus *BvgAS* enables expression of virulence factors in the Bvg^+^ phase. Separate genes are activated to enable phage motility in the environmental, avirulent Bvg^−^ phase [8], [9], [10]. To maintain infectivity, the BP diversity generating retroelement actively mutates the DNA sequence encoding the C-terminus of the Mtd (Fig. 1). During phage propagation, the phage produces a BP-SMPL consisting of a vast library of Mtd variants. A subset of Mtd variants allows the BP to switch tropism, and bind to the new phase of the host. The BP-derived SMPL allows the phage to maintain its infectivity for a dynamically changing host.

Biosynthesis of the BP-SMPL relies upon a phage-encoded, error-prone reverse transcriptase. Sequence information from a non-coding template region (*tr*) of the phage genome is transferred to the variable region (*vr*) at the 3′ terminus of the *mtd* gene, which encodes the C-terminus of the Mtd. Before this transfer, *Bordetella* reverse transcriptase mutates the *tr* mRNA, substituting adenines with any of the four DNA bases [5], [7], [11]. These adenine-dependent mutations correspond to twelve codons located in the coding *vr* region. In its native form, the BP-SMPL is theoretically capable of encoding up to 9.2×10^12^ unique Mtd variants (Fig. 1B).

**Figure 2:**
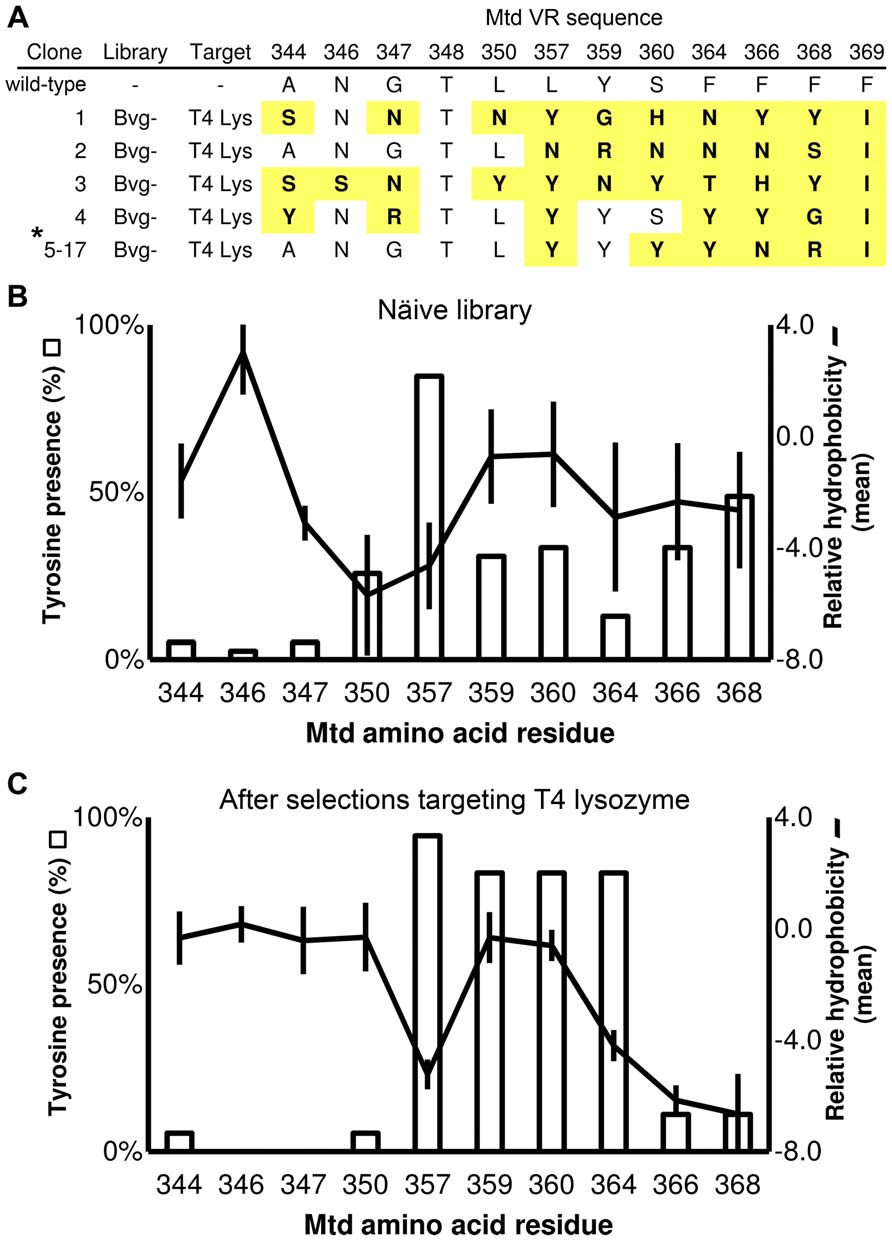
Mtd sequences and selections. (A) The mutable residues of clones recombinantly expressed as GST fusion proteins. The lysozyme-binding L-Mtd variant is indicated by the asterisk. Shading identifies altered residues based on the wild-type Mtd sequence. Analysis of sequences from the (B) naïve Bvg**^–^-**SMPL library and sequences from (C) selections targeting lysozyme. The bar graph indicates the occurrence of tyrosine residues amongst the Bvg**^–^-**SMPL sequences. The line graph indicates mean hydrophobicity scores for the Bvg**^–^-**SMPL sequences relative to wild-type residues. Error bars represent standard deviation (n = 36).

**Figure 3:**
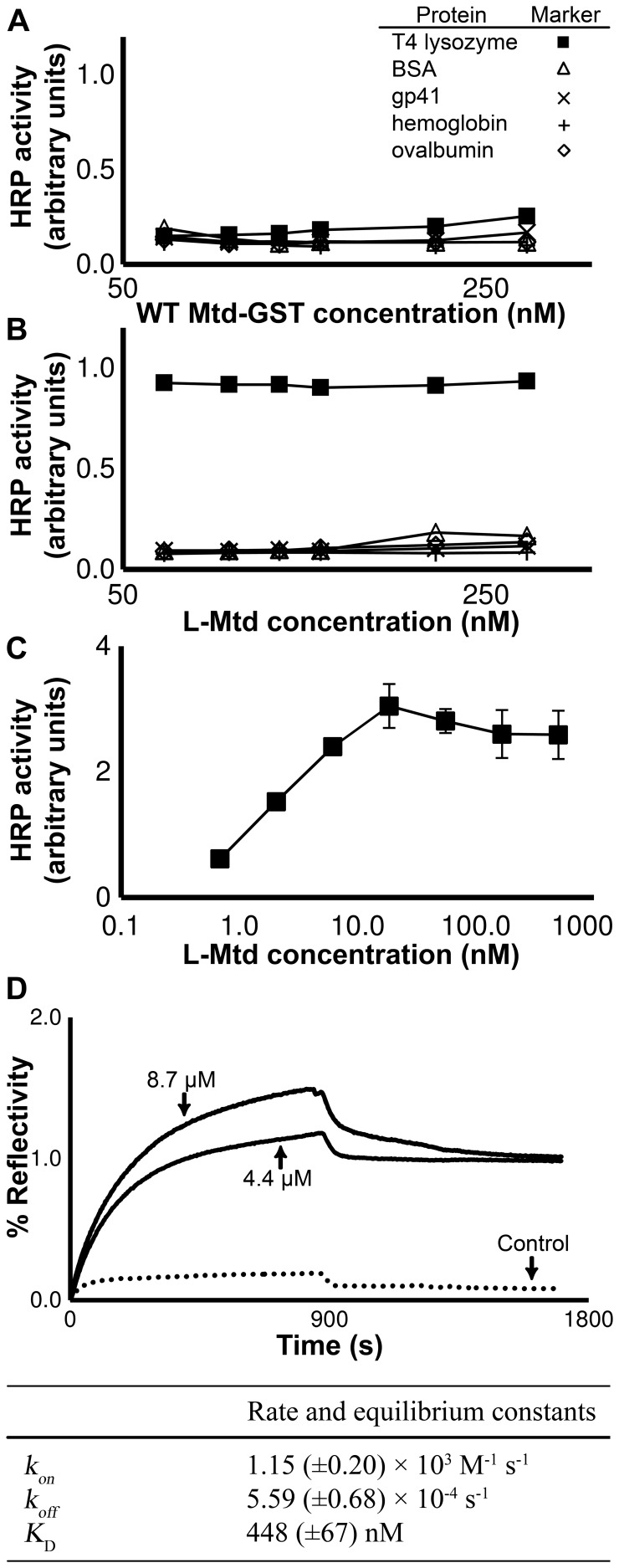
Dose-dependent binding by the recombinantly overexpressed and purified wild-type Mtd and L-Mtd to lysozyme and control proteins. (A) ELISA experiments demonstrate that wild-type Mtd at the indicated concentrations on the X-axis fails to bind to the targeted proteins (coated at 200 nM concentrations) (n = 4). (B) L-Mtd at the indicated concentrations on the X-axis, however, binds with high affinity and specificity to lysozyme and not to the other targeted proteins (coated at 200 nM concentrations) (n = 4). (C) An ELISA repeated on a separate microtiter plate with lower L-Mtd protein concentrations avoids saturation of HRP activity, and demonstrates the high affinity binding by L-Mtd for lysozyme. Error bars in A, B and C depict standard deviation (n = 4). (D) Binding experiments using surface plasmon resonance imaging (SPRI) to measure the affinity of L-Mtd for lysozyme or BSA (negative control). This assay measures the percent change in reflectivity as L-Mtd binds to lysozyme (solid line) or BSA (dotted line) conjugated to the gold layer (solid line). In the table, the rate and dissociation constants were calculated by a least squares fit to the SPRI data with the standard deviation indicated in parentheses (n = 6, single experiment shown).

The BP-SMPL offers tremendous library diversity in a more expedient format than conventional molecular display techniques due to the virus self-synthesizing a new protein library upon propagation in the bacterial host. The bias inherent to propagation could be either avoided or exploited, if characterized for different hosts. Here, we define the properties of SMPLs propagated in two hosts (Fig. 1), and conduct selections targeting T4 lysozyme (subsequently referred to as lysozyme) with libraries produced by the BP (Fig. 2). Protein binding assays with an expressed and purified variant, the lysozyme-binding Mtd (termed L-Mtd) from the BP-SMPL demonstrate the effectiveness of the BP-SMPL system for the identification of specific, high affinity binding partners (Fig. 3).

## Materials and Methods

### Generation of BP-SMPLs

To acquire sufficient phage for selections, four cycles of infection were completed to generate the two BP*-*SMPLs. In the first cycle, a single colony of Bvg^+^ phase bacteria containing the BP prophage was cultured in 3 ml of 2×LB for 16 h at 37°C and shaken at 225 rpm. The culture was centrifuged, and the collected supernatant was filtered through a 0.2 µm pore filter. The soft-overlay method was used to isolate viable phage and to quantify phage titers [12]. Each propagation cycle was repeated using the filtered phage from the previous cycle (3 ml collected and filtered for each cycle). The second round of propagation in Bvg^+^ phase bacteria further increased phage titers. For the third propagation, the filtered BP were divided into two aliquots to infect either Bvg^+^ phase bacteria or Bvg^−^ phase bacteria. The two separate BP*-*SMPLs were re-propagated one additional time in their respective host bacteria to further boost phage titers (Fig. 1C). Further details about the propagation of the BP-SMPLs are provided in File S1.

### Selections with SMPLs

Selections for binding to lysozyme isolated members of the BP-SMPL with low off-rates, using an adaption of previous phage display experiments [13]. Experiments with lysozyme used a pseudo-wild-type variant of lysozyme (a gift from Brian Matthews, University of Oregon) with the following mutations designed to introduce a single cysteine residue into the protein: C54T, S90C, and C97A. Following overexpression and purification of the lysozyme variant by cation exchange chromatography (linear gradient, 0.01 to 1 M NaCl, 20 mM Tris, pH 7.5), the protein was incubated in the reducing agent tris(2-carboxyethyl)phosphine hydrochloride (200 µM), followed by covalent attachment to biotin through incubation with maleimide-PEG_2_-biotin (0.5 mM, Thermo Scientific) [14] for 2 h on ice. The biotinylated lysozyme was then dialyzed into PBS with 0.2% BSA and 0.05% Tween 20 before incubation with phage libraries from the Bvg^+^-SMPL and Bvg^–^-SMPL for 1 h. Next, 100 molar excess non-biotinylated lysozyme (5 µM) was added to the lysozyme - phage library solution (50 nM) for 40 minutes to remove weakly bound phage variants. Capture of the lysozyme-bound phage was accomplished by adding streptavidin-coated paramagnetic beads (0.5 mg, Invitrogen) to the solution. The beads were incubated for five min before rinsing seven times with wash buffer (PBS, 0.2% BSA, 0.05% Tween 20). Phage that remained bound to the magnetic beads were amplified by PCR for DNA sequencing (as described further in File S1). DNA sequences and primers have been submitted to GenBank (accession number pending, submission ID: 1590838).

### Mtd Gene Isolation and Expression of Mtd-GST

After selection, the *mtd* genes of the phage were amplified by standard PCR protocols using primers with encoded *BamHI* and *XhoI* restriction sites (listed in Table S1 in File S1). Standard sequencing methods were used to prepare DNA for sequencing by the Genewiz DNA Sequencing Service. PCR amplicons of the *mtd* gene were ligated into the pGEX-6P-3 expression vector (GE Healthcare), and the resultant plasmid was sub-cloned into a heat shock competent *E. coli* strain (Top Ten) before rescue by addition of SOC media (2% tryptone, 0.5% yeast extract, 8.56 mM NaCl, 2.5 mM KCl, 10 mM MgCl_2_, 20 mM glucose) for 50 min at 37°C. The rescued *E. coli* were then spread on LB plates supplemented with carbenicillin (50 µg/ml) [15].

### Mtd-GST Protein Purification

Mtd variants were transformed and overexpressed in BL21 *E. coli* bacteria as Mtd-GST fusion proteins after induction with isopropyl β-D-1-thiogalactopyranoside (IPTG, 1 mM) for 16 h at 22°C with shaking at 150 rpm. Cell lysate from a 1 L culture was centrifuged at 5000 rpm (3468×g) for 30 min, at 10°C. The cell pellet was reconstituted in 20 ml lysis buffer (500 mM NaCl, 50 mM phosphate, 10 mM imidazole, 1 mM Halt protease inhibitor from Pierce, 10 mM 2-mercaptoethanol, pH 8.0), and sonicated in 30 s continuous bursts with 1 min cooling times for six cycles (20 watts). The sonicated solution was then centrifuged, and the supernatant was applied to an equilibrated GST-Bind resin (3 ml bed volume, Novagen) for 30 min at room temperature. The column was washed with five bed volumes of GST wash buffer (43 mM Na_2_HPO_4_, 14.7 mM KH_2_PO_4_, 1.37 M NaCl, 27 mM KCl, pH 7.3). The protein was eluted with glutathione reconstitution buffer (500 mM Tris-HCl, 10 mM reduced glutathione, pH 8.0). The cell pellet and soluble fractions were analyzed by SDS-PAGE (12% acrylamide).

Mtd-GST protein and lysozyme were further purified by size exclusion chromatography according to manufacture protocols following dialysis into PBS with 3000 MWCO dialysis tubing (BioRad). Overnight cultures of 1 L routinely yielded over 8 mg of purified protein for both wild-type and L-Mtd variants (Table S3 in File S1).

### ELISA Binding Studies

ELISAs were used to examine binding to lysozyme, BSA, hemoglobin, gp41, and ovalbumin by purified, recombinant L-Mtd, wild-type Mtd, and four additional Mtd variants selected for binding to lysozyme. Maxisorp 96-well microtiter plates were coated with 200 µl of the target protein solution (200 nM, unless otherwise noted, in 50 nM NaCHO_3_, pH 9.6), blocked with 0.2% non-fat milk (NFM) in PBS, rinsed with wash buffer and incubated with the Mtd-GST fusion protein variants (1 µM in blocking buffer) for 1 h. The microtiter plates were then incubated with polyclonal rabbit anti-Mtd antibody (1∶500) and HRP-conjugated anti-rabbit antibody (1∶5000) in blocking buffer for 1 h each. The ELISA was developed by the addition of 1% w/v *o*-phenylenediamine dihydrocholoride in citric acid buffer (0.02% w/v H_2_O_2_, 50 mM citric acid, 50 mM Na_2_HPO_4_, pH 5.0), and the absorbance was measured at 450 nM using a microtiter plate reader.

### Surface Plasmon Resonance Biosensor Experiments

Surface plasmon resonance imaging (SPRI) experiments were performed as previously described [16] using a SPRimager II biosensor instrument with SpotReady pure gold sensor chips (GWC Technologies). Protein targets (lysozyme and BSA at 10 µM) were immobilized on the 1-ethyl-3-(3-dimethylaminopropyl)carbodiimide/*N*-hydroxysuccinimide treated sensor chips (0.3 µl in PBS, 1 mM MgCl_2_, pH 8.0) for 1 h. The sensor chip was then blocked with 0.5% NFM in the same buffer (0.8 µl). All measurements were preceded by conditioning the sensor chip with flowing buffer for 30 s. One ml of Mtd-GST solution (8.7 or 4.4 µM in running buffer) was flowed across the sensor chip at 1 µl/s. The increase in pixel intensity for each spot was averaged at 30 frames per s for 900 s. All measurements were conducted in triplicate with subtraction of the background measured for spots treated only with blocking buffer.

## Results and Discussion

### SMPL Production

The synthesis of a BP-SMPL from prophage integrated into the host Bvg^+^ cells requires one or more cycles of phage infection, growth, and isolation. Each cycle can accumulate diverse Mtd sequences for potential tropism switching. Increasing the number of phage in the naïve library provides a more diverse starting population, as the SMPL relies on the host for library biosynthesis. BP produced from the Bvg^+^ wild-type prophage were first propagated for two cycles in Bvg^+^ phase bacteria to increase the viral titers (Fig. 1C).

Following the two cycles of infection and growth in Bvg^+^ bacteria to boost titers, two different SMPLs were generated by infecting either Bvg^+^ or Bvg**^−^** host cells. Each library was then amplified for an additional two cycles to accumulate diversity before selections. Two unique SMPLs resulted from propagation in either the Bvg^+^ or Bvg^−^ bacteria, and were designated as either a Bvg^+^-SMPL or a Bvg^–^-SMPL, respectively. During the four initial cycles of growth in phase-locked strains of *B. bronchiseptica*, the two SMPLs were not subjected to *in vitro* selections, and were thus considered naïve libraries.

To characterize the two initial libraries, the *mtd* genes of naïve BP*-*SMPLs were PCR-amplified and sequenced. The majority of Mtd variants identified from the naïve Bvg**^−^**-SMPL possessed unique *vr* sequences. In contrast, all clones sequenced from the naïve Bvg^+^-SMPL were wild-type (Table S2 in File S1). The lack of diversity in the Bvg^+^-SMPL was expected, as typically only 1 in 10^6^ of the propagated phage can acquire mutations enabling the switch in tropism from Bvg^+^ to Bvg^−^ [17]. Amongst the 47 clones from the naïve Bvg**^−^**-SMPL, 36 unique variations of the Mtd were observed (Table S2 in File S1).

Since SMPLs are biosynthesized, the library volume, phage titers, and percent variation determine the diversity of the library. The diversity of conventional phage display libraries, by comparison, is determined by the transformation efficiency of library DNA into *E. coli* bacteria. The naïve Bvg**^−^**-SMPL produced 3 ml of phage with a titer of 1.2×10^8^ plaque forming units (PFU) per ml to yield an estimated diversity of 3.6×10^8^ Mtd variants (Table S4 in File S1). The titers and consequent library diversity could be expanded considerably using large-scale growth conditions.

### Selections with the BP-SMPLs

To demonstrate the effectiveness of BP-SMPL for the identification of high affinity binders, selections targeted T4 lysozyme. Our lab uses this protein in single molecule studies [18], [19], and its binding partners and inhibitors could provide useful tools for biophysical studies. The Bvg^–^ **-**SMPL was biopanned against biotinylated lysozyme in solution before capture with streptavidin-coated magnetic beads. Following short target incubation and bead capture times, competing non-biotinylated lysozyme (100-fold molar excess) was added to the solution to remove BP variants with fast off-rates. The selections for slow off-rates targeted a single cysteine variant of lysozyme biotinylated through conjugation to biotin-*N*-maleimide. PCR of the selected BP variants followed by sequencing of the *mtd* gene identified five unique Mtd variants. The selectant termed L-Mtd (for lysozyme binding Mtd) comprised 77% of the variants from the off-rate based selections. The results demonstrate convergence on a selectant that is energetically favorable for binding in solution (Fig. 2A).

### Accessing a Tyrosine-rich Library with Mtd Variants

Antibody complementarity-determining regions (CDRs) include flexible loops for the recognition of antigens; this structural flexibility can readily accommodate diverse binding partners within an otherwise rigid immunoglobulin domain recognition [20]. The Mtd features a VR segment in which flexible loops compose roughly half the mutable residues [21]. While not structurally similar to antibodies, the Mtd also provides a versatile system for adaptive molecular recognition with a balance of structural rigidity, sequence diversity, and sufficient malleability, as demonstrated by the ability of the BP-SMPL to generate ligands to novel protein targets not normally encountered by the *Bordetella* phage. Such features are attractive for the identification of binding partners to new targets.

The amino acid tyrosine comprises up to 25% of the complementarity determining regions of functional antibodies [20]. The useful role of tyrosine as a relatively inflexible, hydrophobic, yet potentially hydrogen bonding sidechain has also been demonstrated in the M13 phage display of antibody Fab domains extensively substituted with a “binary code” of exclusively tyrosine and serine residues [22]. Tyrosine residues also enhance the effectiveness of other phage-displayed, Fab libraries [23]. The enrichment for tyrosine substitutions also occurs in the Mtd variants isolated through selections for their slow off-rates for binding to lysozyme.

For example, the L-Mtd has a high percentage of tyrosine residues clustered in the center of the solvent-exposed Mtd VR. L-Mtd and other selectants from the Bvg**^–^-**SMPL leveraged a high percentage of tyrosine residues at position 359, 360, and 364 (Fig. 2C). The L-Mtd variant retains the wild-type VR sequence from positions 344 to 350, and a hydrophilic amino acid substitution profile characterizes positions 364, 366, and 368 (Fig. 2A). For further analysis of the binding properties of selectants from the Bvg^–^ **-**SMPL, a number of Mtd variants, including L-Mtd, were overexpressed and purified without incorporation into phage particles.

### Characterization of Mtd-GST Fusion Proteins

The wild-type Mtd and five Mtd selectants for binding to lysozyme were expressed as fusion proteins to glutathione S-transferase (GST) before purification and biophysical assays. The wild-type Mtd variant contains the sequence from the original prophage, and provides a negative control for the binding studies. The Mtd-GST fusions were purified by glutathione affinity chromatography to >95% homogeneity, as estimated by SDS-PAGE (Fig. S1A in File S1). The most abundant clone from the BP selections, L-Mtd, demonstrated the highest apparent binding affinity of the over-expressed and purified Mtd variants, as measured by ELISA (Fig. S2 in File S1). The dominance of the L-Mtd variant in screens suggests the selection conditions successfully isolated strong binders from the Bvg**^–^-**SMPL. Thus, further biophysical characterization focused on L-Mtd.

Size-exclusion chromatography of the wild-type Mtd and L-Mtd fusion proteins demonstrates that the overexpressed and purified fusion proteins form hexamers *in vitro* (Fig. S1C, D in File S1). This hexameric state could result from GST-mediated dimerization of the Mtd trimer, which has also been observed for other proteins [24]. Notably, cryo-electron microscopy imaging with gold-labeled Mtd on the surface of BP also suggests that the Mtd forms a hexamer on the BP tail fibers [25]. Alternatively, if the Mtd is expressed as a trimer on the tail fiber, similar to other *Podoviridae* bacteriophages, the Mtd-GST would still correctly present the Mtd as a homotrimer, but fused to an additional Mtd trimer.

### Characterization of L-Mtd Binding to Lysozyme

The selectant from the library, L-Mtd, and the negative control, wild-type Mtd, were next examined for binding to lysozyme and additional control proteins. In an ELISA, the wild-type Mtd had no affinity for lysozyme or four control proteins (Fig. 3A). The L-Mtd variant, however, binds with high affinity to the lysozyme target in a dose-dependent manner (Fig. 3B, C). L-Mtd does not bind to BSA, HIV gp41, hemoglobin or ovalbumin, which demonstrates the specificity of the L-Mtd selectant. An additional ELISA with decreased concentrations of lysozyme validates the high affinity interaction between L-Mtd and lysozyme (Fig. 3C).

To further characterize binding affinity, SPRI was used to assay binding by L-Mtd to immobilized lysozyme and BSA (negative control). The target proteins were conjugated covalently to a self-assembled monolayer on the gold surface. The change in percent reflectivity over time allows quantification of the kinetics for L-Mtd binding to either lysozyme or BSA. SPRI measurements demonstrate that the L-Mtd - lysozyme interaction has a dissociation constant, *K*_D_, of 448±67 nM. As expected from the ELISA experiments, L-Mtd does not bind to BSA, the negative control (Fig. 3D). Interestingly, the L-Mtd to lysozyme interaction slows the off-rate by over two orders of magnitude compared to the off-rate of the wild-type Mtd binding to pertactin, at 5.59×10^−4^ s^−1^ and 3.41×10^−2^ s^−1^, respectively [26]. Decreasing the off-rate is pivotal to robust protein-protein binding as the intrinsic on-rate constant rarely exceeds 5×10^6^ M^−1^ s^−1^ for protein-protein interactions [27], [28].

Presenting multiple ligands in a complex can increase the binding affinity through an avidity effect. Previous experiments have demonstrated that the interaction between the wild-type Mtd and pertactin is increased 10^6^-fold when the Mtd is expressed as part of the whole *Bordetella* phage [26]. The weaker monovalent interactions for each Mtd can be amplified by an avidity effect at both the protein scale, with the hexameric Mtd conformation, and at the whole phage scale, with six individual Mtd-containing tail fibers [26]. This potential for multiple levels of avidity could be exploited to obtain large numbers of initial leads, which could be matured into high affinity monomeric receptors. However, the avidity effect requires appropriate geometry between subunits to amplify the apparent receptor-ligand binding affinity. Further experiments are required to determine if avidity effects contribute to the L-Mtd – lysozyme binding interaction reported here.

### Bordetella Bacteriophage as a Lead Generation System

Tropism switching introduces a large difference in diversity between the naïve Bvg^+^- and the Bvg^–^ **-**SMPLs. Binding a new host receptor to infect the Bvg**^−^** bacteria requires the Bvg^+^-derived phage to undergo mutagenesis and tropism switching. Thus, the clones from the naïve Bvg**^–^-**SMPL included abundant mutations in the *vr* of the *mtd* (Table S2 in File S1). Furthermore, the high occurrence of tyrosine residues suggests that the diversity accessed by the Bvg**^–^-**SMPL is well-suited for identifying new binding partners. Though a high percentage of mutations were observed only in the naïve Bvg^–^ **-**SMPL, the L-Mtd variant was isolated after selections from both the Bvg^+^-SMPL and Bvg**^–^-**SMPL libraries. This enrichment ideally reflects the L-Mtd’s high affinity for the target, but could also result from cross-contamination or other trivial reason (e.g., much more vigorous growth by BP displaying L-Mtd). Significantly, the L-Mtd variant was never observed in the naïve libraries (Table S2 in File S1), which demonstrates the effectiveness of the selection conditions. The specific, high affinity binding to a novel target demonstrated by the L-Mtd attests to the robustness of the BP-SMPL.

The approach presented here benefits from the high mutation rate and solvent-exposed accessibility of the BP Mtd VR to uncover unique binding partners to target proteins. However, all molecular display systems are subject to important caveats. Conventional phage display, for example, can be finicky in operation. Other mutagenesis approaches, such as growth in XL1 Red *E. coli* can introduce mutations throughout a plasmid [29]. Generation of the Bvg**^–^-**SMPL offers practical advantages by solving the library synthesis challenge and offering a highly diverse, tyrosine-rich library targeted to a single region of a specific ORF. Growth of the *B. bronchiseptica* Bvg*^+^* bacteria requires Biosafety Level 2 precautions, analogous to tissue culture experiments. We recommend working with the avirulent Bvg**^−^** bacteria, which can be used outside a tissue culture hood. As demonstrated here the Bvg**^–^-**SMPL obtained from the avirulent strain also has greater sequence diversity for selections than the Bvg^+^-SMPL. However, mutagenesis is based on phage propagation, and multiple rounds of selection are not possible unlike conventional M13-based libraries. In addition, adenine substitution of the AAC, ACG, and ATC codons in the native *mtd tr* does not allow mutagenesis to codons encoding the amino acids glutamate, glutamine, lysine, methionine, and tryptophan [21].

Engineered, non-immunoglobin protein scaffolds draw inspiration from antibody-based molecular recognition [30], [31], [32], [33], [34]. For example, like antibodies, the BP-SMPL provides malleable loops on the surface of the protein, which can be adapted to recognize different binding partners; such loops are analogous to the CDR regions of antibodies. In addition to its self-synthesis of protein libraries, the BP-SMPL includes other desirable attributes for a non-immunoglobulin protein scaffold, such as high protein yields from over-expression. Binding ligands identified from selections with the BP-SMPL could be useful in biosensor and single-molecule studies [18], [19], [35], [36].

The dominance of tyrosine residues in the Mtd selectants reported here suggests that results obtained with minimally substituted antibody libraries [22], [37] could be generalized to other adaptable binding scaffolds. This bias for tyrosine substitutions likely increased the potential for obtaining strongly binding receptors. Thus, selections with the tyrosine-rich SMPL from Bvg**^−^** can reduce the time and costs associated with library-based selections. Additionally, prior experiments to enable endogenous mutagenesis of genes heterologous to the *mtd vr* suggest that *Bordetella* phage can expedite directed evolution of a wide variety of protein targets [6], [38]. The high mutation rates generated within the *mtd vr* and the ability to modify adenine-encoded mutational hot spots provides a system for automated mutagenesis of introduced foreign sequences. The approach could overcome the diversity barriers set by the efficiency of bacterial transformation, which is inherent to conventional phage display systems.

## Supporting Information

File S1 Supporting Information file contains Table S1–S4, Figure S1–S2 and Supplemental Materials and Methods.
(DOC)
