# Supplementary material for: Protein Engineering with Biosynthesized Libraries from Bordetella bronchiseptica Bacteriophage
Source: PLoS One. 2013 Feb 7;8(2):e55617. doi: 10.1371/journal.pone.0055617 (PMC3567102; doi:10.1371/journal.pone.0055617)
Supplement: File S1 — Supporting Information file contains Table S1–S4, Figure S1–S2 and Supplemental Materials and Methods. (DOC) [file pone.0055617.s001.doc]

Supporting Information for

**Protein Engineering with Biosynthesized Libraries from *Bordetella bronchiseptica* Bacteriophage**

**Tom Z. Yuan^1^, Cathie M. Overstreet^1^, Issa S. Moody^1^,**

**and Gregory A. Weiss^1,2,3^**

^1^Departments of Molecular Biology and Biochemistry and ^2^Chemistry, University of California, Irvine, California, USA 92697-2025

^3^To whom correspondence should be addressed.

Gregory A. Weiss

University of California, Irvine

Department of Chemistry

1102 Natural Sciences 2

Irvine, CA 92697-2025

USA

Phone: (949) 824-5566

Fax: (949) 824-8571

E-mail: gweiss@uci.edu

**Table of Contents:**

Supplemental Table S1 (p. 2)

Supplemental Table S2 (p.3)

Supplemental Table S3 (p. 4)

Supplemental Table S4 (p. 4)
Supplemental Fig. S1 (p. 5)

Supplemental Fig. S2 (p. 6)

| | Supplemental Materials and Methods (p. 7) **Primer Table** | | | | --- | --- | --- | | *Name* | *Sequence* | *Restriction sites* | | *GstMtdBamHTruncFwd* | CGC GGA TCC AGT ACC GCA GTC CAG TTC CGC | BamH1 | | *MtdMutXhoIRev* | GAC CTC GAG TCA CTA CTC AAG AAT CAG GTG GTC ACA GAC | XhoI | | *VR-Fwd* | TGT AAA ACG ACG GCC AGT TAG CAC TTT GTC GCT TCC | - | | *VR-Rev* | CAG GAA ACA GCT ATG ACT GGC GCA TCC GAA TAC AC | - | |  |  |  |
| --- | --- | --- | --- | --- | --- | --- | --- | --- | --- | --- | --- | --- | --- | --- | --- | --- | --- | --- | --- | --- | --- |

**Supplemental** **Table S1.** Primers utilized for *vr* and *mtd* gene sequencing. Restriction sites were added to allow sub-cloning into the pGEX-6P-3 vector (GE Healthcare).

| |  |  | ***mtd vr* Position** | | | | | | | | | | | |  | | --- | --- | --- | --- | --- | --- | --- | --- | --- | --- | --- | --- | --- | --- | --- | | **Variant** | **Library** | 344 | 346 | 347 | 348 | 350 | 357 | 359 | 360 | 364 | 366 | 368 | 369 | # of clones | | Wild-type | - | A | N | G | T | L | L | Y | S | F | F | F | F |  | | 1 | Bvg^+^-SMPL | A | N | G | T | L | L | Y | S | F | F | F | F | 17 | | 18 | Bvg^-^-SMPL | S | S | N | T | T | Y | R | N | N | Y | Y | I | 1 | | 19 | Bvg^-^-SMPL | S | P | N | T | N | Y | L | S | N | Y | Y | I | 1 | | 20 | Bvg^-^-SMPL | S | S | N | T | I | Y | T | D | G | N | Y | F | 1 | | 21 | Bvg^-^-SMPL | S | S | N | T | S | L | Y | S | Y | F | Y | L | 1 | | 22 | Bvg^-^-SMPL | S | S | N | T | N | Y | Y | S | Y | N | Y | I | 1 | | 23 | Bvg^-^-SMPL | A | S | N | T | N | Y | N | D | D | P | Y | I | 1 | | 24 | Bvg^-^-SMPL | A | S | N | T | A | Y | Y | N | F | F | F | F | 1 | | 25 | Bvg^-^-SMPL | S | S | R | T | N | S | Y | A | N | Y | G | I | 1 | | 26 | Bvg^-^-SMPL | S | T | N | T | N | Y | D | Y | A | A | Y | I | 1 | | 27 | Bvg^-^-SMPL | S | S | R | T | N | Y | N | N | S | G | G | V | 1 | | 28 | Bvg^-^-SMPL | S | S | N | T | S | Y | N | N | F | F | F | F | 1 | | 29 | Bvg^-^-SMPL | A | S | N | T | Y | Y | Y | S | F | F | F | F | 1 | | 30 | Bvg^-^-SMPL | A | S | N | T | Y | Y | Y | T | F | F | F | F | 1 | | 31 | Bvg^-^-SMPL | S | S | N | T | H | Y | N | I | L | Y | F | L | 1 | | 32 | Bvg^-^-SMPL | S | A | Y | T | Y | Y | A | S | S | A | N | V | 1 | | 33 | Bvg^-^-SMPL | Y | S | R | T | N | Y | N | S | N | S | G | I | 1 | | 34 | Bvg^-^-SMPL | S | S | N | T | H | Y | N | H | N | A | Y | I | 1 | | 35 | Bvg^-^-SMPL | Y | N | R | A | Y | Y | N | N | N | Y | G | I | 1 | | 36 | Bvg^-^-SMPL | S | A | Y | T | N | Y | N | S | S | A | N | I | 1 | | 37 | Bvg^-^-SMPL | S | S | R | T | Y | Y | N | Y | N | Y | Y | I | 1 | | 38 | Bvg^-^-SMPL | F | N | N | T | N | Y | N | N | Y | Y | Y | I | 1 | | 39 | Bvg^-^-SMPL | S | S | N | T | Y | Y | G | S | F | F | F | F | 3 | | 42 | Bvg^-^-SMPL | S | L | N | T | P | Y | Y | Y | Y | T | Y | I | 1 | | 44 | Bvg^-^-SMPL | A | S | N | T | N | Y | N | N | F | F | F | F | 7 | | 53 | Bvg^-^-SMPL | A | S | N | T | N | Y | H | Y | F | F | F | F | 1 | | 54 | Bvg^-^-SMPL | A | S | N | T | L | L | Y | T | F | F | F | F | 1 | | 55 | Bvg^-^-SMPL | A | S | N | T | L | L | N | S | F | F | F | F | 1 | | 56 | Bvg^-^-SMPL | A | S | N | T | N | Y | Y | Y | T | S | F | I | 1 | | 57 | Bvg^-^-SMPL | A | Y | N | T | N | Y | S | Y | N | Y | F | I | 1 | | 58 | Bvg^-^-SMPL | A | N | N | A | N | Y | S | Y | F | A | Y | I | 1 | | 59 | Bvg^-^-SMPL | S | S | N | T | Y | F | S | Y | A | Y | Y | I | 1 | | 60 | Bvg^-^-SMPL | S | S | N | T | Y | Y | S | Y | N | L | Y | I | 1 | | 61 | Bvg^-^-SMPL | S | S | N | T | N | Y | N | Y | A | Y | Y | I | 1 | | 62 | Bvg^-^-SMPL | S | S | N | T | Y | Y | S | I | F | Y | Y | I | 1 | | 63 | Bvg^-^-SMPL | S | S | N | T | Y | Y | Y | Y | N | Y | Y | I | 1 | | 64 | Bvg^-^-SMPL | A | L | N | T | N | N | Y | Y | S | Y | Y | I | 1 | |  |  |  |
| --- | --- | --- | --- | --- | --- | --- | --- | --- | --- | --- | --- | --- | --- | --- | --- | --- | --- | --- | --- | --- | --- | --- | --- | --- | --- | --- | --- | --- | --- | --- | --- | --- | --- | --- | --- | --- | --- | --- | --- | --- | --- | --- | --- | --- | --- | --- | --- | --- | --- | --- | --- | --- | --- | --- | --- | --- | --- | --- | --- | --- | --- | --- | --- | --- | --- | --- | --- | --- | --- | --- | --- | --- | --- | --- | --- | --- | --- | --- | --- | --- | --- | --- | --- | --- | --- | --- | --- | --- | --- | --- | --- | --- | --- | --- | --- | --- | --- | --- | --- | --- | --- | --- | --- | --- | --- | --- | --- | --- | --- | --- | --- | --- | --- | --- | --- | --- | --- | --- | --- | --- | --- | --- | --- | --- | --- | --- | --- | --- | --- | --- | --- | --- | --- | --- | --- | --- | --- | --- | --- | --- | --- | --- | --- | --- | --- | --- | --- | --- | --- | --- | --- | --- | --- | --- | --- | --- | --- | --- | --- | --- | --- | --- | --- | --- | --- | --- | --- | --- | --- | --- | --- | --- | --- | --- | --- | --- | --- | --- | --- | --- | --- | --- | --- | --- | --- | --- | --- | --- | --- | --- | --- | --- | --- | --- | --- | --- | --- | --- | --- | --- | --- | --- | --- | --- | --- | --- | --- | --- | --- | --- | --- | --- | --- | --- | --- | --- | --- | --- | --- | --- | --- | --- | --- | --- | --- | --- | --- | --- | --- | --- | --- | --- | --- | --- | --- | --- | --- | --- | --- | --- | --- | --- | --- | --- | --- | --- | --- | --- | --- | --- | --- | --- | --- | --- | --- | --- | --- | --- | --- | --- | --- | --- | --- | --- | --- | --- | --- | --- | --- | --- | --- | --- | --- | --- | --- | --- | --- | --- | --- | --- | --- | --- | --- | --- | --- | --- | --- | --- | --- | --- | --- | --- | --- | --- | --- | --- | --- | --- | --- | --- | --- | --- | --- | --- | --- | --- | --- | --- | --- | --- | --- | --- | --- | --- | --- | --- | --- | --- | --- | --- | --- | --- | --- | --- | --- | --- | --- | --- | --- | --- | --- | --- | --- | --- | --- | --- | --- | --- | --- | --- | --- | --- | --- | --- | --- | --- | --- | --- | --- | --- | --- | --- | --- | --- | --- | --- | --- | --- | --- | --- | --- | --- | --- | --- | --- | --- | --- | --- | --- | --- | --- | --- | --- | --- | --- | --- | --- | --- | --- | --- | --- | --- | --- | --- | --- | --- | --- | --- | --- | --- | --- | --- | --- | --- | --- | --- | --- | --- | --- | --- | --- | --- | --- | --- | --- | --- | --- | --- | --- | --- | --- | --- | --- | --- | --- | --- | --- | --- | --- | --- | --- | --- | --- | --- | --- | --- | --- | --- | --- | --- | --- | --- | --- | --- | --- | --- | --- | --- | --- | --- | --- | --- | --- | --- | --- | --- | --- | --- | --- | --- | --- | --- | --- | --- | --- | --- | --- | --- | --- | --- | --- | --- | --- | --- | --- | --- | --- | --- | --- | --- | --- | --- | --- | --- | --- | --- | --- | --- | --- | --- | --- | --- | --- | --- | --- | --- | --- | --- | --- | --- | --- | --- | --- | --- | --- | --- | --- | --- | --- | --- | --- | --- | --- | --- | --- | --- | --- | --- | --- | --- | --- | --- | --- | --- | --- | --- | --- | --- | --- | --- | --- | --- | --- | --- | --- | --- | --- | --- | --- | --- | --- | --- | --- | --- | --- | --- | --- | --- | --- | --- | --- | --- | --- | --- | --- | --- | --- | --- | --- | --- | --- | --- | --- | --- | --- | --- | --- | --- | --- | --- | --- | --- | --- | --- | --- | --- | --- | --- | --- | --- | --- | --- | --- | --- | --- | --- | --- | --- | --- | --- | --- | --- | --- | --- | --- | --- | --- | --- | --- | --- | --- | --- | --- | --- | --- | --- | --- | --- | --- | --- | --- | --- | --- |

**Supplemental** **Table S2.** Mtd sequences of naïve Bvg^+^-SMPL and naïve Bvg^-^-SMPL variants. The yellow highlighting indicates deviation from the wild-type prophage sequence.

| *Variant* | *mg/ml* | *Volume (ml)* | *Yield (mg)* |
| --- | --- | --- | --- |
| Wild-type Mtd | 6.327 | 1.50 | 9.49 |
| L-Mtd | 5.541 | 1.50 | 8.31 |

**Supplemental** **Table S3.** Protein yields for 1 L of overexpressed wild-type Mtd and L-Mtd (1 mM IPTG, 37 °C, 225 rpm, 16 h) after purification by GST affinity chromatography and size exclusion chromatography.

| Library | Theoretical diversity | Titers (PFU/ml) | Practical diversity |
| --- | --- | --- | --- |
| *Bordetella* phage | 9.2 x 10^12^ | 1.2 x 10^8^ | 3.6 x 10^8^ |
| M13 phage protein library | Varies | 10^8^ - 10^10^ | Up to 10^10^ |
| PCR-driven library construction (Antibody sourced) | Up to 10^9^ | - | Up to 10^9^ |
| Kunkel mutagenesis | Up to 10^10^ | - | Up to 10^10^ |

**Supplemental Table S4.** Practical and theoretical diversities from BP-SMPL production and comparable M13 libraries [Kehoe JW, Kay BK (2005) Filamentous phage display in the new millennium. Chem Rev 105: 4056-4072].


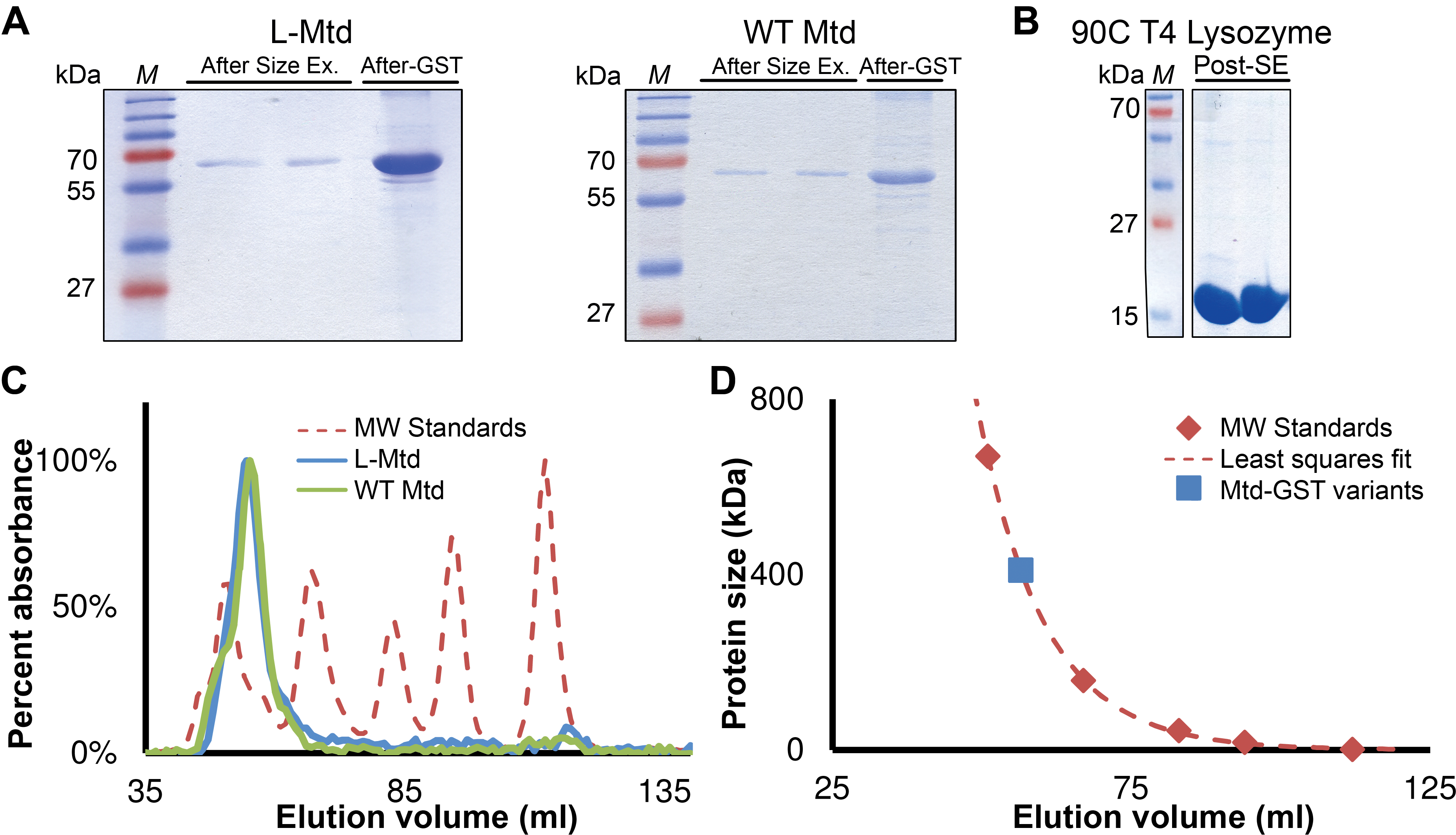


**Supplemental** **Fig. S1.** Characterization of recombinantly expressed and purified (A) Mtd–GST fusion protein and (B) 90C T4 lysozyme. Mtd-GST variants were purified by GST affinity chromatography followed by size exclusion (SE) chromatography. The T4 lysozyme was purified by cation exchange chromatography. (C) The Mtd-GST fusion proteins were purified by size exclusion chromatography, which was calibrated with MW standards (dashed line, Bio-Rad). (D) A least squares fit to an exponential decay model of the data from size exclusion with MW standards allows estimation of protein size as a function of elution volume. The measured MW of approximately 410 kDa approaches the theoretical size of the Mtd-GST hexamer (406 kDa).

**
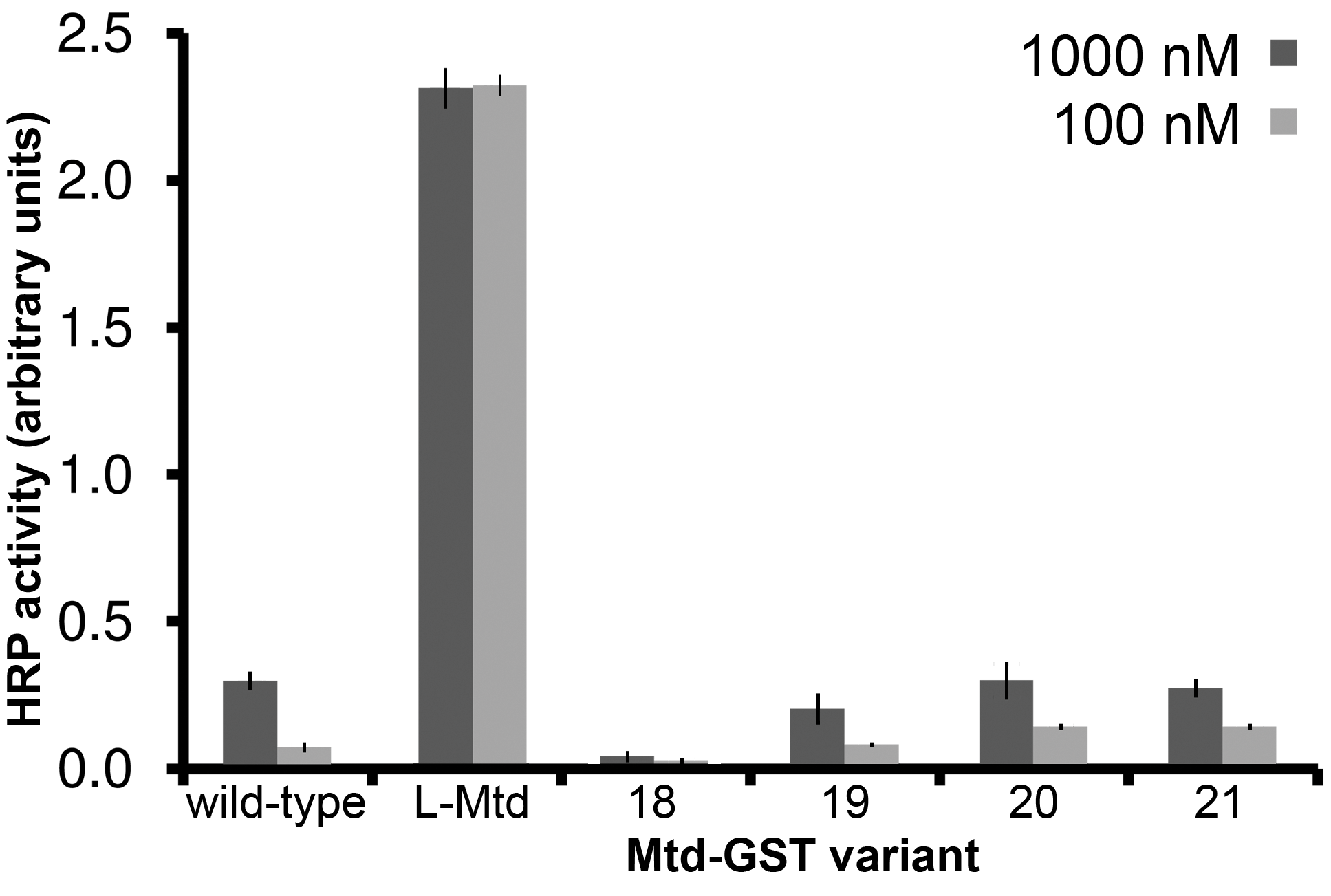
**

**Supplemental Fig. S2** The L-Mtd receptor isolated from the BP SMPL binds specifically to T4 lysozyme. In this ELISA, lysozyme has been adsorbed to a microtiter plate at the indicated concentration. L-Mtd expressed expressed as a Mtd-GST fusion protein binds with greater affinity to lysozyme than other selectants from the library. Error bars indicate standard deviation (n=4).

**Supplemental Materials and Methods**

*Generation of BP-SMPLs*

Four cycles of infection were completed to generate the Bvg^+^- and Bvg^-^-SMPLs. In the first cycle, a single colony of Bvg^+^ phase bacteria containing the BP prophage was cultured in 3 mL of 2x LB (20 g tryptone, 10 g yeast extract, 5 g NaCl, 1 l H_2_O, pH 7.0) for 16 h at 37 °C and shaken at 225 rpm. The culture was transferred to two Eppendorf tubes, and centrifuged at 12 krpm for 5 min at room temperature. The supernatant was filtered (0.2 µm) to obtain pure BP. The following soft-overlay method was used to isolate viable phage. Briefly, 115 µl of the filtered supernatant and 230 µl of log-phase Bvg^+^ phase bacteria were added to 3 mL of 42 °C 0.7% w/v top agar (0.7 g agar, 100 mL LB). The mixture was vortexed briefly and then poured over a pre-warmed 115x10 mm LB agar plate (10 g tryptone, 5 g yeast extract, 10 g NaCl, 15 g agarose, 1 L H_2_O) containing streptomycin (40 µg mL^-1^) before incubating for 16 h at 37 °C. BP particles were removed from the translucent plate by adding 3 mL of SM buffer (5.8 g NaCl, 2 g MgSO_4_·7 H_2_O, 5 mL 2% w/v gelatin, 1 L H_2_O) followed by incubation at 4 °C for 3 h on a rotary shaker with gentle rocking. After incubation, the supernatant containing viable phage was removed from the plates and filtered. The soft overlay method using 50 µl of either Bvg^+^ or Bvg^-^ phase bacterial cells was used to generate titer plates supporting a lawn of bacteria on LB plates containing streptomycin (40 μg mL^-1^). The supernatant containing viable phage was diluted in 10-fold dilutions in SM buffer and titered on LB-streptomycin plates. Plates were incubated for 18 h at 37 °C, and the plaque forming units counted.

Each propagation cycle was repeated using the filtered phage from the previous cycle. The second propagation in Bvg^+^ phase bacteria further increased phage titers. For the third propagation, the filtered BP were divided into two aliquots to infect either Bvg^+^ phase bacteria or Bvg^-^ phase bacteria. The two separate SMPLs were re-propagated one additional time in the respective host bacteria to boost phage titers. The SMPLs were normalized to 1.2 x 10^8^ PFU mL^-1^ by dilutions in SM buffer.

*Identification and sequencing phage after selections*

PCR was used to amplify 3 μl of eluted phage in a 28 μl reaction (17 µl H_2_O, 5.6 µl 5x FlexiBuffer buffer (Promega), 1.5 µl 50 mM MgCl_2_, 0.25 µl 25 mM dNTPs, 0.3 µl (5U/µl) GoTaq polymerase (Promega), 0.25 µl 330 ng/µl *VR-Fwd*, 0.25 µl 330 ng/µl *VR-Rev*). The reaction was cycled in a thermocycler (95 °C for 5 min, 38 x (95 °C for 1 min, 65 °C for 30 s, 72 °C for 1 min), and then 72 °C for 7 min).
